# Deep Paleoproteotyping and Microtomography Revealed No Heart Defect nor Traces of Embalming in the Cardiac Relics of Blessed Pauline Jaricot

**DOI:** 10.3390/ijms24033011

**Published:** 2023-02-03

**Authors:** Virginie Bourdin, Philippe Charlier, Stéphane Crevat, Lotfi Slimani, Catherine Chaussain, Mélodie Kielbasa, Olivier Pible, Jean Armengaud

**Affiliations:** 1 Museum of Quai Branly—Jacques Chirac, 222 rue de l’Université, 75007 Paris, France; 2 Laboratory Anthropology, Archaeology, Biology (LAAB), UFR of Health Sciences (Paris-Saclay University), 2 avenue de la Source de la Bièvre, 78180 Montigny-Le-Bretonneux, France; 3 Foundation Anthropology, Archaeology, Biology (FAAB)—Institut de France, 23 quai de Conti, 75006 Paris, France; 4 Arlliage, 6 Grande Route des Feuillants, F-69001 Lyon, France; 5 Université Paris Cité, Laboratory URP2496 Orofacial Pathologies, Imaging and Biotherapies, PIV Platform, Dental School, 1 rue Maurice Arnoux, 92120 Montrouge, France; 6 APHP, Hôpital Bretonneau, GH Nord Université Paris Cité, 75018 Paris, France; 7 Université Paris-Saclay, CEA, INRAE, Département Médicaments et Technologies pour la Santé (DMTS), SPI, 30200 Bagnols-sur-Cèze, France

**Keywords:** forensic anthropology, paleopathology, metaproteomics, mass spectrometry, proteotyping, micro-CT scan, relics, cardiac characterization

## Abstract

Scientific examination of the heart of Blessed Pauline Jaricot—a French missionary figure—was carried out in 2022. As tandem mass spectrometry proteotyping has proven to be valuable to obtain the broad taxonomic repertoire of a given sample without any a priori information, we aimed at exploring the conditions of preservation of the relics and possible conditions of death. Metaproteomics and high-resolution microtomography imaging approaches were combined. A dataset comprising 6731 high-resolution MS/MS spectra was acquired and 968 of these spectra could be assigned to specific peptidic biomolecules. Based on the taxonomical information encompassed by the identified peptide sequences, 5 phyla were identified amongst eukaryota (94% of the biomass): Ascomycota (55%), with the species *Aspergillus versicolor*, *Trichophyton mentagrophytes* and *Aspergillus glaucus*, corresponding to expected cadaverous fungal flora; Chordata (42%), represented by a unique species, *Homo sapiens*; Streptophyta (3%); and Arthropoda (traces). Bacteria (6% of the biomass) were poorly represented. No trace of embalming substance could be retrieved, nor any pathogens. Imaging evidenced no heart defect nor embalming traces. No evidence that was inconsistent with natural and spontaneous conservation could be retrieved. This study prefigures the power of modern molecular techniques such as paleoproteotyping coupled to microtomography to gain insight into historical relics.

## 1. Introduction

As religion and miracles have always been shrouded in a veil of mystery, many have examined and still question the authenticity of relics presented as holy. Macroscopic examination provides valuable information about the pathologies that may have affected the health of concerned individuals. For example, the examination of the mandible of Saint-Louis—one of the most famous French kings—allowed the identification of the relics and improved our knowledge about the circumstances of the king’s death [1]. While many bone examinations are now based on paleoradiology [2], comprehensive investigations most often involve forensic anthropologists, medical examiners, pathologists, geneticists, radiologists, biochemists, palynologists, zoologists and archaeologists [3,4]. The use of radiocarbon dating, e.g., in the case of the Holy Shroud of Turin, one the most studied relics [5,6], and of computer-aided facial reconstruction [7] are other supportive techniques. Paleoproteomics, the study of ancient proteins, has succeeded in taxonomically identifying bones, authenticating foods, and characterizing pathogens in order to explain past disease [8]. Tandem mass spectrometry proteotyping has recently proven to be a rapid method to obtain the broad taxonomic repertoire of a given sample without any a priori information [9]. Such methodologies could deliver novel insights into historical relics. 

Pauline-Marie Jaricot was born into a prosperous family of silk workers on 21 July 1799, in Lyon. At age 16, a sermon she heard in Saint-Nizier, her parish, put her back on her true path: she burnt romantic books and passionate songs, abandoned her jewelry, disposed of her most beautiful dresses to dress like a proletarian [10] (p. 16) and vowed to remain chaste in body and heart [10] (p. 17). That Pauline Jaricot has always remained canonically secular is all the more remarkable because non-religious, unmarried women were poorly considered at that time [11] (p. 7). She is famously known for having founded the work of the Propagation of the Faith, intended to collect money for missionary works [10] (p. 24), which was a success. At the same time, she founded the faith preservation charity called the Living Rosary, a vast enterprise of communion of prayers which, for France alone, collected 2,250,000 subscribers in 1862 [10] (p. 48). The Living Rosary still exists, animated by the Dominicans of Lyon, and has spread as far as the Pacific Islands. At the end of the 20th century, it was still practiced in Tahiti [10] (p. 48).

Pauline Jaricot suffered from poor health her entire life. Mention is made of a “most characteristic aneurysm, complicated by a disease of a nature unknown to science” which left her “not enough blood in her veins so that […] one could explain the extension of her life” [12] (p. 47). Quite ill, she went to Rome in 1835, and Pope Gregory XVI honored her with a visit. Writings describe her as very weak, livid and suffering from painful palpitations at that time [12] (pp. 60–61). Against all odds and about two weeks after her meeting with the Pope, Pauline was healed in Mugnano (in northwest Naples, Italy) by her faith in Saint Philomena, the relics of whom she brought back to Lyon and to the vicar of Ars [10] (pp. 55–56). Swollen legs and feet have been mentioned in writings in the years following this episode [12] (p. 114). Pauline Jaricot died on 9 January 1862, in Lyon [13]. For the last two months of her life, she was particularly ill [12] (p. 321). She was coughing up blood for the last few days of her life, and a wound had formed on her chest [12] (pp. 330–331). Pauline Jaricot’s heart was removed at death.

In 1889, the heart was solemnly taken to the archdiocese of Lyon and then to the church of Saint-Polycarpe in Lyon. It has been resting there, in the Saint François-Xavier chapel, ever since [10] (p. 99). In 1935, Pauline Jaricot’s remains, which rested in the family vault in the historic cemetery of Loyasse, were transported to the Saint-Nizier church [10] (p. 100). On 25 February 1963, Pope John XXIII declared her “Venerable” [10] (p. 101). 

In 2012—Pauline Jaricot’s jubilee year, marking the one hundred and fiftieth anniversary of her death—three-year-old Mayline Tran lost consciousness after choking while eating. The child, hospitalized in Lyon in a desperate state after several cardiovascular arrests, was in an irreversible coma and her situation was considered beyond recovery; a novena to Pauline Jaricot was initiated. Against all odds, Mayline Tran experienced a healing considered remarkable and unexpected [14]. On 26 May 2020, the Vatican recognized the miracle, and Pauline Jaricot was beatified on 22 May 2022, in Lyon.

On the occasion of the restoration of the cardiotaph containing the heart of Pauline Jaricot, and in the context of the beatification process, we were asked by the Archbishopric of Lyon (France) to perform a comprehensive scientific examination of her mummified heart. We planned to analyze the conditions of preservation of the heart and determine whether post mortem care included embalming. We then wanted to explore the heart disease hypothesis as a possible cause of death.

## 2. Results

### 2.1. Macroscopic Examination

**Figure 1:**
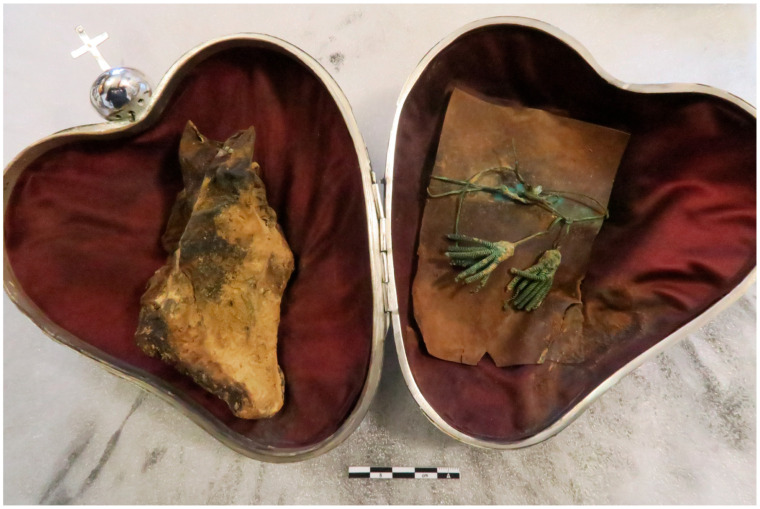
Heart of P. Jaricot at the opening of the silver cardiotaphe; on the right, cellulosic element (cardboard) and cardinal tassels.

The heart was still quite identifiable, with certain anatomical areas recognizable (Figure 1). The side facing the upper shell of the reliquary showed deposits of adipocere, a process of transformation of certain organic tissues which contributes to their long-term preservation.

There were declive dark brown spots or slicks, that we believe to be fluids rather than fungal deposits, as they permeate the fabric in depth. We interpreted the spots as dried blood, as the heart was placed fresh in the reliquary. These traces were located on the back side of the heart, which was in contact with the lower shell of the reliquary. These deposits also impregnated certain areas of the velvet sheathing the interior of the reliquary and, in particular, those in contact with the heart; this could indicate that the heart was enclosed in the reliquary immediately following its removal, as decomposition was about to begin, which was then stopped by rapid drying. However, no sampling could be performed for further analysis, as it would have been destructive for the relic.

### 2.2. Micro-CT Scan

**Figure 2:**
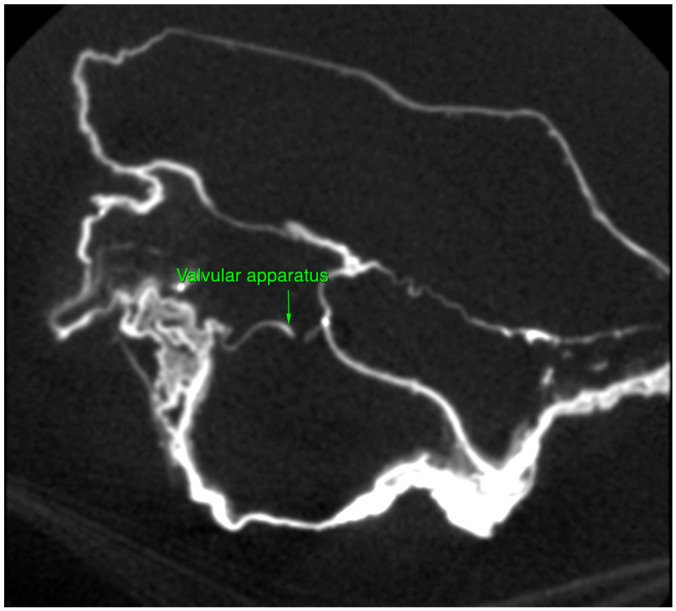
Collapsed heart valve, heart of P. Jaricot.

Micro-CT analysis confirmed the cardiac nature of the organ by direct observation of four cavities, large vessels and valvular apparatus (Figure 2).

**Figure 3:**
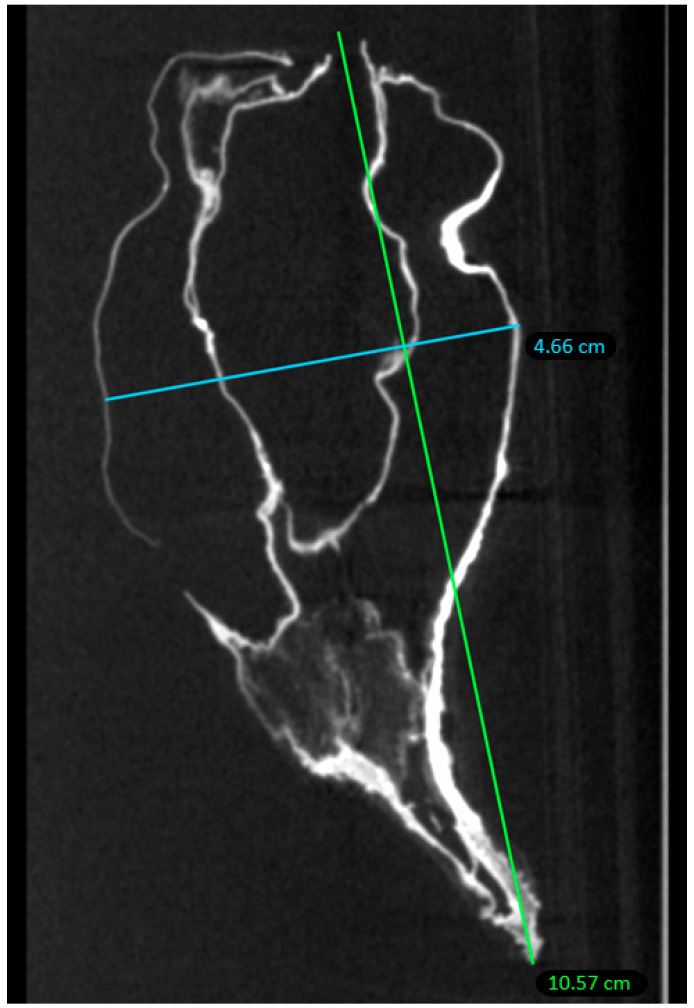
Axial measurements of the heart of P. Jaricot.

Within the conservation limits of this organ, the heart exhibited human morphology: a 10.6 cm long axis and 4.7 cm short axis, within the section limits of the examination (Figure 3). It was retracted and deformed on its external part; the interior was morphologically preserved.

**Figure 4:**
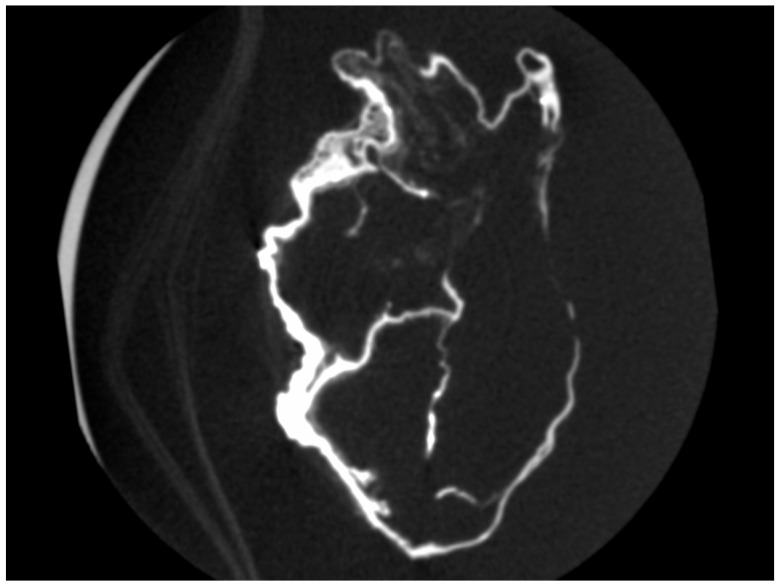
Four cavities of the heart of P. Jaricot.

**Figure 5:**
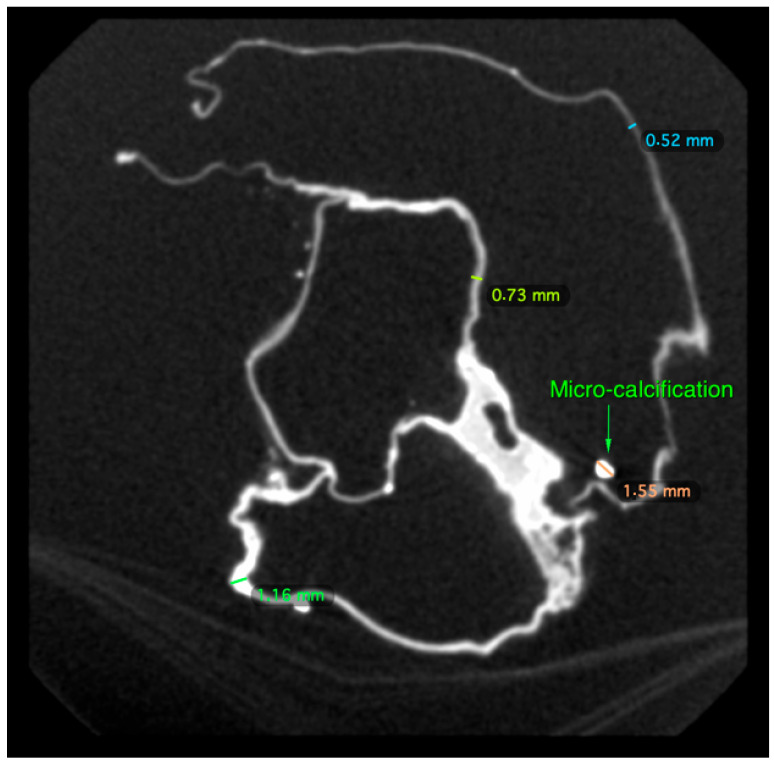
Parietal micro-calcification and measurement of the external/inter-cavitary walls of the heart of P. Jaricot.

There was an overall and diffuse thinned appearance of all the cardiac walls and four cavities, including at the level of the departure of the large vessels, which is probably of post mortem and non-pathological origin; the thickness of the outer wall was between 0.5 and 4 mm, often less than 1 mm; the thickness of the inter-cavity walls was between 0.5 and 1 mm (Figure 4 and Figure 5). 

We spotted the presence of numerous parietal micro-calcifications and of a few rare intraluminal ones, probably of taphonomic origin (Figure 5); these calcifications, frequently observed on decomposed or putrefied corpses in forensic medicine, are the result of cadaveric processes. Mineral precipitation by the activity of living organisms—so-called biomineralization—is a process that occurs from bacteria to chordates [15]. The mineral formation takes place in various animals in a process in which the organism produces an organic framework to introduce ions for further crystallization and growth mediated by an organic matrix [16]. Microorganisms release metabolites, which favor the modification of the local environment by increasing the pH and elevating the supersaturation, which results in the precipitation of minerals. Additional frameworks include some macromolecules and cell structures that can act as heterogeneous crystallization nuclei, inducing precipitation [17,18]. The different types of minerals that bacteria can produce include nitrates, silicates, calcium oxalates, halides, apatite, gypsum, oxides, phosphates and calcium carbonate [17,19]. A similar taphonomic process of precipitation of ionic elements (calcium, magnesium, phosphorus) present in the blood, in the lymphatic fluid and in the cytoplasm “sweating” from myocardial dead tissues and large vessels at their base (aorta, superior and inferior vena cava, pulmonary artery) has been described in the heart of French–Polish musician Frederic Chopin [20]. 

We could not find any coronary thrombus, or atheroma lesion—usually very well conserved post mortem, especially when calcified—nor any evidence of a dilated or hypertrophic heart disease, of arrhythmogenic dysplasia of the right ventricle (the thinning, in this case, was complete and not localized in a particular area of the organ) or of pulmonary arterial hypertension.

**Figure 6:**
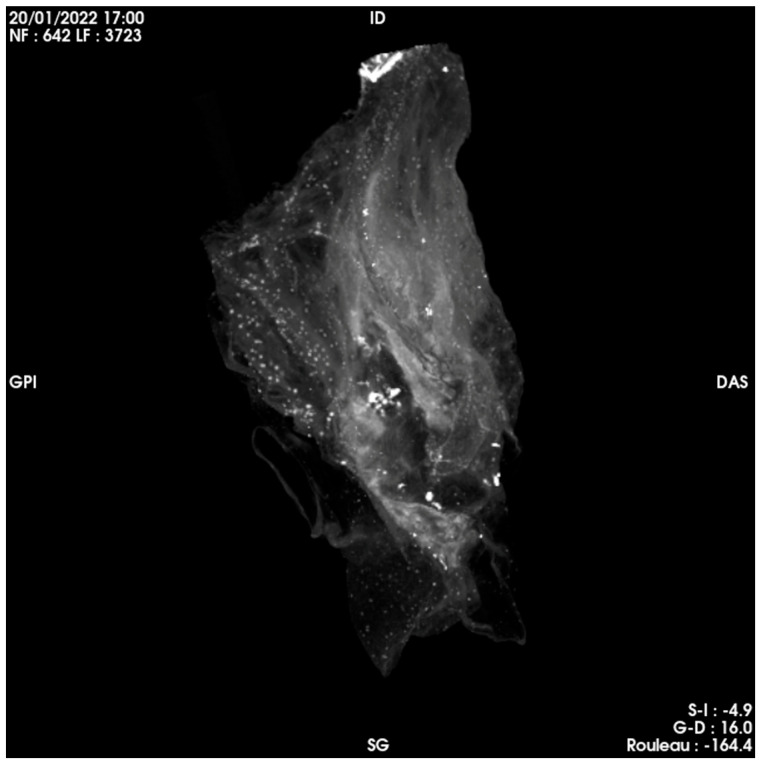
Three-dimensional reconstruction of the heart of Pauline Jaricot.

Unlike the heart of Anne-Madeleine Remuzat [21], the outside of which had been brushed with honey, and the four cavities of which had been opened on their side, emptied of blood clots and filled with herbs and fragrant plants, there was no trace of opening in this case. There was no trace of filling or sections other than aortic and/or pulmonary arteries (these cavities being optically empty). Finally, there did not appear to be any distemper nor coating of the exterior walls. Three-dimensional reconstruction of the organ from the scanning imagery is shown in Figure 6.

### 2.3. Tandem Mass Spectrometry Proteotyping

Tandem mass spectrometry proteotyping allows the identification of organisms based on peptide sequences established, without any a priori information, by metaproteomic query of a generalist protein sequence database [22]. For this, proteins extracted from the sample were proteolyzed and the resulting peptides were analyzed by tandem mass spectrometry. A total of 6,731 high-resolution MS/MS spectra were acquired on peptides in a unique dataset. This is a relatively low number, because 10 times more spectra can be obtained under similar conditions for a sample rich in proteins, but the initial sample was available in a very small quantity. The spectra were queried to identify the possible organisms present in the sample at the different possible taxonomical ranks, using the same procedure as previously used for medical samples without a metagenomics-derived database [9]. The wide taxonomical repertoire was obtained through a cascade of searches, starting from the extra-large NCBInr database and narrowing down successively to the most representative database for the specific sample. The final query resulted in peptide sequence assignment for 968 MS/MS spectra, i.e., 14% of spectra interpreted. Taxonomical information was assigned to each of these spectra; i.e., the list of taxa whose genome encodes a given peptide sequence was established at all possible taxonomical ranks. The results are displayed in Table 1. The distribution of the resulting 357 peptide sequences and 968 taxon-spectrum matches (TSMs) on the different branches of the Tree of Life indicated the presence of biological material originating from Eukaryota and Bacteria. A total of 301 peptides specifically found in Eukaryota theoretical proteomes and 25 peptides specific to Bacteria were identified. The respective biomass ratio was estimated from the TSMs assigned at the superkingdom rank: 910 for Eukaryota and 58 for Bacteria; i.e., 94% and 6%, respectively. 

#### 2.3.1. Identification of the Eukaryota Taxa Evidenced by Metaproteomics-Based Proteotyping

Five eukaryotic phyla could be identified based on taxon-specific peptides: Ascomycota (55% of the protein biomass based on TSMs), Chordata (42%), Streptophyta (3%), Arthropoda (trace amount). The first three phyla were detected unambiguously with 128, 88 and 14 phylum-specific peptides, respectively. Among the abundant fungi, 2 genera were distinguished with the following representative species: *Aspergillus versicolor* (28 species-specific peptides, 78% of the fungus biomass), *Trichophyton mentagrophytes* (24 species-specific peptides, 14% of the fungus biomass), and *Aspergillus glaucus* (13 species-specific peptides, 8% of the fungus biomass). The Chordata phylum was only explained by a unique species, *Homo sapiens*. The traces originating from plants came from a single species related to *Brassica napus*, commonly known as canola or rape. The trace amount related to Arthropoda was related to a unique species: *Leptinotarsa decemlineata* (potato beetle), identified by six species-specific peptides. 

#### 2.3.2. Characterization of Human Proteins

The metaproteomics search revealed the identification of 204 peptide sequences that could point at 130 possible proteins grouped into 123 protein groups. The low level of peptides per protein (average of 1.6) is classical in metaproteomics due to the large diversity of peptides present in the sample, the sampling effect of the nanoLC-MS/MS analysis and the large repertoire of theoretical proteomes used for the assignment [22]. Because of their abundance, human protein sequences are well covered: 70 peptides have been assigned to 28 human proteins (average of 2.5 peptides per protein). Table 2 shows that among the most abundant proteins, the serpin peptidase inhibitor (a protein central in controlling coagulation pathways) is the most detected (85 MS/MS spectra). Other abundant proteins are: hemoglobin (34 MS/MS spectra), beta globin (22 MS/MS spectra), and Titin (13 MS/MS spectra). Titin is a large, abundant protein of striated muscle, which contains binding sites for muscle-associated proteins to serve as an adhesion template for the assembly of contractile machinery in muscle cells; it is mainly synthetized in heart tissue. Several keratins and collagens were also evidenced. Interestingly, neutrophil defensin 3, a protein that has antibiotic, fungicide and antiviral activities, was identified with three unambiguous peptides. 

#### 2.3.3. Identification of the Bacterial Components of the Sample

While Bacteria are poorly represented in the sample, trace amounts of 8 genera could be sketched based on a low number of taxon-specific peptides: Microbacterium (5), Streptomyces (4), Pseudonocardia (3) and Clostridium (3), represented by the species *Microbacterium hydrocarbonoxydans, Streptomyces bungoensis*, *Pseudonocardia thermophila* and *Clostridium transplantifaecale*, respectively. Most of these species are environmental bacteria, and only *Clostridium transplantifaecale*, a recently characterized new species retrieved by extensive culturomics in 2018 from human stool from a patient with recurrent *Clostridium difficile* (currently known as *Clostridioides difficile*) infection [23], is known to be pathogenic.

In conclusion, the metaproteomics-based proteotyping results indicated the presence of fungi, most of them being classical decomposition fungal flora. It also evidenced human tissues, with abundant keratins, serpins and titin, a protein mainly expressed in the heart. Plants and insects were only present in traces. Bacteria were mainly environmental, except for one species commonly retrieved in guts and stool.

## 3. Discussion

Although Pauline Jaricot was described as cardiac with elements that may suggest arrhythmia (palpitations) or heart failure (swollen legs), we did not find here any evidence of heart impairment as a cause of death.

The description of Pauline Jaricot’s last days displayshemoptysis and a foul-smelling breast lesion, suggestive of either a pulmonary and lymph node tuberculosis associated to a skin fistula, a pulmonary aspergillosis or a malignant tumor with pulmonary invasion and terminal hemoptysis. 

The sample has been extensively characterized by metaproteomics, revealing the presence of significant fungal flora, the main representatives of which are *Aspergillus versicolor* (39.2% of the analyzed organic biomass), *Trichophyton mentagrophytes* (7.0%) and *Aspergillus glaucus* (3.8%). Evidence of these three fungal species is not surprising: human remains and other archeological materials are often highly contaminated with microorganisms. Establishing a correlation with a potential diagnosis of aspergillosis is difficult, as the fragments analyzed are fragments that fell to the bottom of the reliquary and most likely correspond to surface deposits; fungal flora develops more particularly on exterior surfaces. Thus, the development of Aspergillus is particularly conditioned by environmental conditions: temperature, relative humidity, duration of sunny periods and agents of air pollution [24,25]. The study of the mummified human remains of three members of the Kuffner family (19th–20th century, Slovakia), including muscles, bones, skin and funeral clothes, allowed for the identification of fungi. Most isolated fungi belonged to the genera Aspergillus and Penicillium [26]. This observation is reproducible: samples of human remains were collected during archaeological works in the vault of the Cathedral Basilica of St. John the Baptist and the Assumption of the Blessed Virgin Mary (Fredro crypt) in Przemyśl (Poland): xerophilic fungi were the most numerous in all samples; fungi belonging to the genera Penicillium and Aspergillus dominated, with the main species being *Penicillium chrysogenum* (36.4%) and *Aspergillus versicolor* (19.7%) [27]. Regarding bacterial isolates, all strains isolated in this Polish study belonged to the genus Bacillus (*pumilus*, *subtilis*, *cereus*) [27]. The same description for Aspergillus strains was reported in five Yemeni mummies (fourth century B.C., National Museum of Yemen, Sana’a) who were studied to evaluate hydrolytic enzymes produced as a result of fungal contamination: forty-seven fungal species were isolated after cultivation, reflecting a certain degree of contamination which may have resulted from the poor ventilation and preservation system. Aspergillus was the most common genus isolated, mainly represented by species *A. niger* and *A. flavus* [28].

*A.versicolor*, which is also able to grow on very nutrient-poor materials such as concrete and plaster [29], and *Trichophyton mentagrophytes* are keratinophylic fungi; *T. mentagrophytes* breaks down keratinous substrates by both chemical and mechanical processes; five different keratinolytic enzymes from ten strains of *T. mentagrophytes* have been isolated [30]. A survey on keratinophilic fungi from poultry-farm and feather-dumping soils in India revealed the existence of 34 species of fungi; *A. versicolor* and *T. mentagrophytes* were efficient candidates, among other fungi, to degrade feathers. *A. glaucus* also proved to be an efficient degrader when cultivated in a medium containing feathers as the sole nutrient source. Among all species, *T. mentagrophytes,* alongside *Scopulariopsis brevicaulis,* produced higher amounts of keratinase in both methods [31]. The quantity of keratins found in our metaproteomics analysis is a factor that can explain the development of fungal strains, at least at the surface of the main biological material and recovered as dust from the cardiotaph. 

In addition to being common cadaverous fungal flora, *A. versicolor* and *A. glaucus* display pathogenicity in both immunodeficient and immunocompetent patients. *A. versicolor* is known to be the cause of a particular nondermatophyte onychomycosis, external auditory canal infections, lung aspergilloma, endophtalmitis following corneal microperforation and asthma discomfort [32,33,34,35,36,37]. Regarding *A. glaucus,* it was evidenced in both a fatal brain infection (cerebral aspergillosis) and a primary cutaneous aspergillosis, both infections affecting immunocompetent patients [38,39]; however, this species is rarely isolated from bronchial secretions or other clinical samples, and invasive infections are also very uncommon [40]. *Aspergillus fumigatus*—retrieved here—is nowadays the pathogen accounting for roughly 90% of invasive mold infections [41,42]; although environmental and health conditions may have been different some 2 centuries ago, we do not believe *A. versicolor* could be considered as a serious pathogen. *T. mentagrophytes* triggers dermaphytosis, such as onychomycosis—also referred to as “ringworm”—in immunocompetent adults. It is currently predominantly of zoophilic origin and often leads to inflammatory tinea (tinea capitis, t`inea corporis or tinea genitalis) [43,44,45,46,47]. However, we do not believe, in this case, that either the *A. versicolor*, *A. glaucus* or *T. mentagrophytes* species may have caused pathogenicity or may have accounted for the death of Pauline Jaricot. Despite being a potential cause of invasive systemic infections, chronic pulmonary aspergillosis and lung aspergilloma in immunococompetent patients, *A. versicolor* has been much more frequently reported in immunocompromised hosts [48]; aspergilloma has also been reported as a complicating factor for pre-existing lung cavities, underlying lung conditions such as chronic obstructive pulmonary disease or sarcoidosis, prior or concurrent tuberculous or non-tuberculous mycobacterial disease [34,48,49]. We have no biological proof to affirm the coexistence of tuberculosis. Post mortem growth of A. versicolor as cadaverous flora is the most likely hypothesis, although we cannot formally eliminate the hypothesis of contamination by contiguity during the extraction of the heart. *A. glaucus* does not display tropism for in vivo cardiac tissue and clearly corresponds to post mortem fungal flora. *T. mentagrophytes* could have been brought by external hands having extracted and manipulated the heart during its original extraction and transport.

Regarding the abundance of keratins—not naturally present in the heart—we believe they come from hands of the surgeon who extracted the heart when Pauline Jaricot died. Only the presence of titin, which is the main component of the longitudinal filaments ensuring the maintenance of the myofibrillar architecture, can confirm the presence of cardiac tissue in the analyzed sample. 

Amongst bacteria, only *Clostridium transplantifaecale* may display pathogenicity and is commonly found in the intestinal tract; because Pauline Jaricot was not reported as suffering from chronic diarrhea, we do not attribute the presence of this species to a particular condition; all the more so as it was retrieved in the heart.

One limit to our analysis is that we worked on fragments found at the bottom of the cardiotaph: sampling the holy relic itself was not an option. Therefore, these debris and dust may comprise mainly the fungal spores that were able to detach from the organ surface, enhancing this specific type of organic biomass, and keratins brought by operators during excision, sampling and cardiotaph insertion. Another limit regarding our results depends on the mass spectrometry proteotyping approach, for which the precision regarding the identified species depends on the number of sequenced genomes available for the closely related species. For example, *T. mentagrophytes* is the best option amongst Trichophyton, but only 14 species have been genome sequenced so far for this genus, while 32 species have been taxonomically defined. As we have used one of the most advanced tandem mass spectrometers currently available for protein analysis, we believe that the resulting metaproteomic dataset is of high quality and could be further reanalyzed in the future once more genome sequences are available. 

In conclusion, it is necessary to emphasize the central place of the heart in the spirituality of Pauline Jaricot, but also in her earthly life, with her miraculous healing in 1835, and the material and moral persecutions she endured at the end of her life, which literally “rended” her heart.

The macroscopic, CT-scan and proteotyping analysis of the heart enabled us to assert the human and cardiac nature of the organ. We could not find any evidence in favor of a cardiac origin for death, nor any element accounting for either the tuberculosis or the tumoral hypotheses. Although lung aspergillosis followed by post mortem fungal dissemination—as the heart was extracted from the chest—remains a possibility, we consider fungi and bacteria retrieved as environmental contamination. Our position is that fungal cultures have developed under particularly favorable conditions of temperature, humidity, pH and nutrient environment. This is surface contamination, not present and not pathogenic in Pauline Jaricot’s lifetime. We could not locate any sign of embalming displaying an anatomical preparation of the organ (no opening nor filling of the organ with exogenous material). No evidence that was inconsistent with natural and spontaneous conservation not mediated by the hand of man, which can be considered a miracle by the Roman Catholic Church, could be retrieved. At last, this study shows the power of modern molecular techniques to gain insight into historical relics. We propose the generic wording “paleoproteotyping” to name the approach of tandem mass spectrometry proteotyping developed in the present study for the analysis of ancient relics and artefacts.

## 4. Materials and Methods

### 4.1. Cardiotaph Description

The reliquary is a silver box, the dimensions of which are 21 × 16.5 × 11 cm (height × width × thickness) consisting of 2 shells forming a heart, joined on 1 side by a hinge. It is surrounded by a wide white satin ribbon sealed with the arms of Cardinal Maurin (Archbishop of Lyon from 1916 to 1936), displaying an engraved inscription “Cor Paulinae Mariae Jaricot exeogitata ab ipsa et ordinata fuit amplissima stipius collectio … Operis Propagationis Fideis Leo PP XIII Bref du 13 juin 1881”.

The heart was extracted from its cardiotaph on 20 January 2022, at the dental school of Université Paris Cité.

### 4.2. Micro-CT Scan

High-resolution micro-CT imaging was obtained using a Quantum FX Caliper micro-CT scanner (Life Sciences, Perkin Elmer, Waltham, MA, USA) hosted by the PIV platform located at laboratory URP 2496 from the dental school of Université Paris Cité (Montrouge, France). The scanning parameters applied for the heart were: tube voltage set at 90 kV and tube current at 160 μA. The scans were performed with a 73 mm field of view with a 148-μm isotropic voxel size (scan time of 34 s), and with a 60 mm field of view with a 118-μm isotropic voxel size (scan time of 36 s). Full 3D high-resolution raw data were obtained by rotating both the x-ray source and the flat panel detector 360° around the heart. Views are reconstructed in a stack of images containing 512 × 512 × 512 voxels. Following the scan, tridimensional images were reconstructed and the resulting images were reoriented in a standardized manner by OsiriX software (Pixmeo, version 5.8.5; Bernex-Switzerland).

### 4.3. Tandem Mass Spectrometry Proteotyping 

Heart debris and dust fallen to the bottom of the cardiotaph were collected and subjected to tandem mass spectrometry proteotyping. In accordance with the request of the Pontifical Missionary Societies, no sampling altering the integrity of the relic was carried out. A quantity of 42.5 mg of debris was subjected to bead-beating in presence of 99 µL of lithium dodecyl sulfate (LDS) 1X lysis buffer using a Precellys Evolution device (Bertin Technologies) to extract proteins, as previously described [50]. Proteins were fully denatured for 5 min at 99 °C. A volume of 20 µL of the resulting sample was subjected to electrophoresis for 5 min on a NuPAGE 4–12% Bis-Tris gel. The proteins were stained with Coomassie SimplyBlue SafeStain (Thermo Fisher Scientific), washed extensively, and then in-gel proteolyzed with trypsin as described [51]. The resulting peptides were analyzed with an Exploris 480 tandem mass spectrometer connected to a Vanquish Neo UHPLC (Thermo-Fisher) with the same parameters as previously described [52]. Briefly, peptides were resolved by reversed-phase chromatography using a long gradient (5%–25% for 90 min of 0.1% HCOOH/100% CH3CN against 0.1% HCOOH/100% H2O, followed by 25%–40% for 5 min) using a 50 cm EasySpray column at a flow rate of 0.250 μL/min. Resolution was set at 120,000 for the parent ions and 15,000 for the secondary ions. A dynamic exclusion time of 10 s was activated. For MS/MS spectra assignment, a cascade search was performed with Mascot Daemon 2.6.1 (Matrix science), as previously described [53], using as parameters full trypsin specificity, maximum of one missed cleavage, 3 ppm mass tolerance on the parent ion, 0.02 Da tolerance on the secondary ions, carboxyamidomethylation of cysteine residues and oxidation of methionine residues as possible modification. For each taxonomical rank and each taxon, the taxon-spectrum matches (TSMs), as previously defined [54], and the number of taxon-specific peptide sequences were quantified. Human proteins that were identified, but with no annotated function, were systematically analyzed with the conserved domain tool from NCBI [55].

### 4.4. Mass Spectrometry Data

The mass spectrometry proteomics data have been deposited with the ProteomeXchange Consortium via the PRIDE partner repository (https://www.ebi.ac.uk/pride/ accessed on 21 January 2023) with the dataset identifier PXD039007 and 10.6019/PXD039007.

## Figures and Tables

**Table 1:** Taxonomical assignment by tandem mass spectrometry proteotyping.

SK	TSMs	spePEPs	PHYLUM	TSMs	spePEPs	CLASS	TSMs	spePEPs	ORDER	TSMs	spePEPs
Eukaryota	910	301	**Chordata**	**367**	**88**	**Mammalia**	**370**	**88**	**Primates**	**370**	**88**
			Ascomycota	487	128	Eurotiomycetes	487	128	Eurotiales	429	55
									Onygenales	56	24
			Streptophyta	29	14	Magnoliopsida	29	14	Brassicales	31	14
			Arthropoda	0	6	Insecta	24	6	Coleoptera	24	6
Bacteria	58	25	Actinobacteria	26	14	Actinobacteria	23	14	Pseudonocardiales	11	3
									Micrococcales	8	5
									Streptomycetales	4	4
			Firmicutes	0	6	Bacilli	0	3	Bacillales	6	3
						Clostridia	7	3	Clostridiales	7	3
			Proteobacteria	0	1	Alphaproteobacteria	15	1	Rhodobacterales	15	1
			Bacteroidetes	0	4	Flavobacteriia	7	4	Flavobacteriales	7	4
			**FAMILY**	**TSMs**	**spePEPs**	**GENUS**	**TSMs**	**spePEPs**	**SPECIES**	**TSMs**	**spePEPs**
			**Hominidae**	**370**	**88**	**Homo**	**370**	**88**	***H. sapiens***	**374**	**88**
			Aspergillaceae	429	55	Aspergillus	429	55	*A. versicolor*	378	28
									*A. glaucus*	37	13
			Arthrodermataceae	58	24	Trichophyton	58	24	*T. mentagrophytes*	68	24
			Brassicaceae	29	14	Brassica	29	14	*B. napus*	29	14
			Chrysomelidae	24	6	Leptinotarsa	24	6	*L. decemlineata*	24	6
			Pseudonocardiaceae	12	3	Pseudonocardia	12	3	*P. thermophila*	12	3
			Microbacteriaceae	7	5	Microbacterium	7	5	*M. hydrocarbonoxydans*	7	5
			Streptomycetaceae	4	4	Streptomyces	4	4	*S. bungoensis*	4	4
			Bacillaceae	2	2	Bacillus	2	2	*B. caseinilyticus*	2	2
			Paenibacillaceae	0	1	Paenibacillus	4	1	*P. albidus*	4	1
			Clostridiaceae	7	3	Clostridium	7	3	*C. transplantifaecale*	7	3
			Rhodobacteraceae	15	1	Rhodobacter	15	1	*R. ovatus*	15	1
			Flavobacteriaceae	7	4	Flavobacterium	7	4	*F. cucumis*	4	1

SK: superkingdom; TSMs: taxon-spectrum matches (null value is assigned to TSMs if all the TSMs at a given taxonomical rank have been assigned to other taxa due to parsimony rule); spePEPs: taxon-specific peptides.

**Table 2:** Human proteins ranked by their abundances, assessed by the number of MS/MS spectra.

Protein Accession	Functional Description	Assigned Peptides	MS/MS Spectra	Protein Group
EAW81588.1	Serpin peptidase inhibitor	11	85	A (8 shared peptides)
CAA25459.1	Serpin-like protein	9	5	A (8 shared peptides)
AQN67653.1	Hemoglobin subunit alpha	2	34	
AAY46275.1	Beta globin chain, partial	3	22	
NP_006112.3	Keratin, type II cytoskeletal 1	9	19	
EAX11019.1	Titin, isoform CRA_e	8	13	
NP_000412.4	Keratin, type I cytoskeletal 10 isoform 1	5	11	B (3 shared peptides)
CAA60378.1	Keratin, type I	2	1	B (3 shared peptides)
XP_005257116.2	Collagen alpha-1(I) chain isoform X3	2	9	
NP_005208.1	Neutrophil defensin 3	3	9	
NP_000217.2	Keratin, type I cytoskeletal 9	4	8	
AAC15854.1	Ribosomal protein S13	1	7	
CAB56534.1	Fibrillin 15	1	7	
AAI28106.1	Histone H4B	2	6	
BAG60658.1	Albumin domain-containing protein	3	5	
AAB59495.1	Alpha-1-antitrypsin	1	4	
EAW56540.1	Rho family protein	1	4	
BAB79461.1	Ribosomal protein L15	1	3	
NP_001952.1	Elongation factor 2	1	3	
AAH42586.1	Collagen, type I, alpha 2	2	3	
ACE96343.1	5-hydroxytryptamine (serotonin) receptor 2B	1	2	
BAD92412.1	Collagen, type V, alpha 3	1	2	
BAG61384.1	Keratin type II	2	1	
NP_001373235.1	Antithrombin-III isoform 7	1	1	
XP_011533341.1	Sacsin isoform X2	1	1	
EAW90166.1	Translation initiation factor 4A, isoform 1	1	1	
NP_001837.2	Collagen alpha-2(IV) chain	1	1	
EAW94269.1	Proteasome 26S subunit, ATPase 5	1	1	

## Data Availability

The mass spectrometry proteomics data have been deposited with the ProteomeXchange Consortium via the PRIDE partner repository (https://www.ebi.ac.uk/pride/ accessed on 21 January 2023) with the dataset identifier PXD039007 and 10.6019/PXD039007.
